# Longitudinal associations between late-life depression dimensions and cognitive functioning: a cross-domain latent growth curve analysis

**DOI:** 10.1017/S003329171600297X

**Published:** 2016-11-11

**Authors:** A. Brailean, M. J. Aartsen, G. Muniz-Terrera, M. Prince, A. M. Prina, H. C. Comijs, M. Huisman, A. Beekman

**Affiliations:** 1 Department of Health Service and Population Research, King's College London, Institute of Psychiatry, Psychology and Neuroscience, Centre for Global Mental Health, London, UK; 2 NOVA - Norwegian Social Research, Center for Welfare and Labor Research, Oslo, Norway; 3 Centre for Dementia Prevention, University of Edinburgh, UK; 4 VU University Medical Centre, Department of Psychiatry and the EMGO Institute for Health and Care Research, Amsterdam, The Netherlands; 5 VU University Medical Center, Department of Epidemiology and Biostatistics and the EMGO Institute for Health and Care Research, Amsterdam, The Netherlands; 6 Department of Sociology, VU University, Amsterdam, The Netherlands; 7 VU University Medical Centre, Department of Psychiatry and the EMGO Institute for Health and Care Research, Amsterdam, The Netherlands

**Keywords:** Cognitive abilities, cognitive ageing, depression symptom dimensions, late-life depression, latent growth curve models

## Abstract

**Background:**

Cognitive impairment and depression often co-occur in older adults, but it is not clear whether depression is a risk factor for cognitive decline, a psychological reaction to cognitive decline, or whether changes in depressive symptoms correlate with changes in cognitive performance over time. The co-morbid manifestation of depression and cognitive impairment may reflect either a causal effect or a common cause, depending on the specific symptoms experienced and the cognitive functions affected.

**Method:**

The study sample comprised 1506 community-dwelling older adults aged ⩾65 years from the Longitudinal Aging Study Amsterdam (LASA). We conducted cross-domain latent growth curve analyses to examine longitudinal associations between late-life depression dimensions (i.e. depressed affect, positive affect, and somatic symptoms) and specific domains of cognitive functioning (i.e. processing speed, inductive reasoning, immediate recall, and delayed recall).

**Results:**

Poorer delayed recall performance at baseline predicted a steeper increase in depressed affect over time. Steeper decline in processing speed correlated with a steeper increase in somatic symptoms of depression over time.

**Conclusions:**

Our findings suggest a prospective association between memory function and depressed affect, whereby older adults may experience an increase in depressed affect in reaction to poor memory function. Somatic symptoms of depression increased concurrently with declining processing speed, which may reflect common neurodegenerative processes. Our findings do not support the hypothesis that depression symptoms may be a risk factor for cognitive decline in the general population. These findings have potential implications for the treatment of late-life depression and for the prognosis of cognitive outcomes.

## Introduction

Extensive evidence suggests that late-life depression co-occurs with cognitive dysfunctions affecting in particular fluid cognitive abilities such as processing speed, executive function, and episodic memory (e.g. Comijs *et al.* 2001; Lockwood *et al.* 2002; Baudic *et al.* 2004; Sheline *et al.* 2006; Morimoto & Alexopoulos, 2013; Koenig *et al.* 2014). However, the direction of influence remains unclear, leaving an unresolved question of whether depression is a risk factor for cognitive impairment, a psychological reaction to cognitive impairment, or a prodromal syndrome of dementia.

To overcome problems of reversed causation, a number of studies have investigated bi-directional prospective associations between depression symptoms and cognitive functioning. Some studies found that higher baseline levels of depressive symptoms predicted steeper decline in general cognitive performance in persons aged 60–80 years (Gale *et al.* 2012), steeper decline in delayed recall and global cognitive function in persons aged 65–84 years (Panza *et al.* 2009), as well as slower processing speed and reaction time at follow-up in persons aged 70–97 years (Bunce *et al.* 2014), whereas baseline cognitive performance did not predict changes in depressive symptoms over time. Other studies found evidence for the opposite direction of the effect whereby poorer attention and episodic memory in persons aged ⩾85 years (Vinkers *et al.* 2004), poorer episodic memory in persons aged ⩾50 years (Jajodia & Borders, 2011), and poorer global cognitive performance in persons aged ⩾70 years (Perrino *et al.* 2008) predicted an increase in depression symptoms over time, whereas baseline depression levels were unrelated to the rate of cognitive decline. A study by van den Kommer *et al.* (2013) found evidence for both directions of the effect whereby higher depression symptoms at baseline predicted steeper decline in processing speed and general cognitive ability over time, and slower processing speed at baseline predicted an increase in depression symptoms over time in persons aged 55–85 years. The same study by van den Kommer *et al.* (2013) found no significant association between the course of depressive symptoms and the course of general cognitive functioning or processing speed. The mixed evidence regarding the longitudinal association between depression symptoms and cognitive functioning in late life may be due to methodological differences between studies such as the number of assessment waves included, the length of follow-ups, the age range of participants, and the cognitive and depression measures included.

Most of the longitudinal studies above measured depression as a unidimensional construct and did not distinguish between different symptom dimensions. However, late-life depression is a heterogeneous condition. Specific symptom presentations such as depressed affect, low positive affect, somatic and motivational, or cognitive symptoms may reflect distinct aetiologies, and may be differentially related to the nature and course of cognitive impairment. For instance, affective symptoms of depression may manifest as a psychological reaction to mild or transitory cognitive impairment, whereas an organic syndrome of depression, consisting of psychomotor and cognitive symptoms, may be more prevalent as cognitive functioning deteriorates and older adults lose insight about their cognitive deficits (Ritchie *et al.* 1999).

Previous reports suggest that motivational and somatic symptoms of depression may be more strongly related to cognitive impairment than affective symptoms. Cross-sectional findings suggest that ‘motivational disturbance’ was more strongly related to verbal fluency performance than ‘affective suffering’ symptoms (Castro-Costa *et al.* 2007; Brailean *et al.* 2015). Longitudinal findings suggest that patients with a history of vascular disease who had poorer baseline performance on executive function, processing speed and memory tasks showed a higher increase in a cluster of motivational and somatic symptoms of depression compared to mood symptoms over 7 years of follow-up (Kooistra *et al.* 2015). These findings are consistent with the depression-executive dysfunction hypothesis according to which motivational symptoms of depression tend to occur in the context of executive deficits, possibly due to vascular disease and a disruption of frontal-subcortical pathways (Alexopoulos *et al.* 1997, 2002). Evidence for the prominent role of motivational and somatic symptoms in persons with cognitive impairment comes also from studies suggesting that symptoms of fatigue, cognitive complaints and sleep disturbance were associated with ‘cognitive impairment no dementia’ in the absence of dysphoric and anhedonic symptoms (Potvin *et al.* 2010), and that motivational symptoms of depression were dominant in the preclinical phase of Alzheimer disease (Berger *et al.* 1999; Bartolini *et al.* 2005), and in mild cognitive impairment (Kumar *et al.* 2006). However, other findings suggest that depressed affect and somatic symptoms were similarly related to cognitive performance on attention and motor tasks in the general population (Baune *et al.* 2007).

Evidence on the association between positive affect and cognitive functioning is also inconclusive. Cross-sectional studies suggest that positive affect was a more robust predictor of cognitive performance across a variety of tasks than depressed affect, somatic symptoms or interpersonal difficulties (La Rue *et al.* 1995), that higher positive affect was related to better every day problem solving (Paterson *et al.* 2016), and that higher positive affect was related to better verbal fluency performance (before Bonferroni adjustment), but not better memory, speed or attention (Baune *et al.* 2007). Longitudinal findings by Turner *et al.* (2015) suggest that lower baseline levels of positive affect (but not somatic symptoms, interpersonal difficulties, or depressed affect scores) were associated with steeper decline in global cognition, episodic memory, and perceptual speed. Positive affect could help maintain cognitive function by reducing stress hormone levels and cardio-vascular risk, by improving health behaviours such as diet, sleep, and physical exercise, and by increasing the engagement in social interactions and cognitively stimulating activities (for a review see Pressman & Cohen, 2005).

Studies employing a longitudinal design and a multidimensional approach to depression are needed to clarify the direction of influence between depression symptom dimensions and cognitive functioning, and to help gain a better understanding of the neurobiological and psychological mechanisms underlying the co-morbid manifestation of depression and cognitive impairment in late-life. Our study aims to examine longitudinal associations between specific depression symptom dimensions (i.e. depressed affect, positive affect, somatic symptoms) and specific domains of cognitive functioning (i.e. processing speed, inductive reasoning, immediate recall, delayed recall) in older adults. If depression symptoms develop as a psychological reaction to cognitive impairment, we would expect lower baseline cognitive functioning to predict an increase in depression symptoms over time. This effect may be stronger for affective symptoms of depression. If depression symptoms are a risk factor for cognitive impairment, we would expect higher initial depression levels to predict steeper cognitive decline. This effect may be stronger for somatic symptoms of depression. If cognitive impairment and depression symptoms share a similar aetiology (e.g. dementia-related neuropathology, cerebrovascular diseases) we would expect a synchronous relationship whereby an increase in depression symptoms would correlate with declining cognitive function over time. This effect is more likely to be observed for somatic symptoms of depression. The simultaneous examination of these hypotheses could help clarify the effect magnitude and direction of influence between cognitive abilities and late-life depression dimensions. For comparison purposes we include also an examination of the longitudinal associations between cognitive abilities and depression conceptualized as a unitary construct (i.e. total CES-D score).

## Method

### Participants

**Fig. 1.**
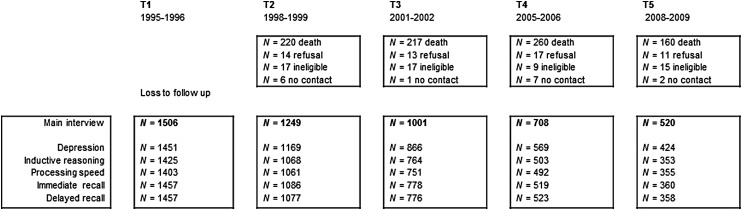
Flow chart of the study sample.

Data were used from the Longitudinal Aging Study Amsterdam (LASA; Huisman *et al.* 2011), an ongoing study exploring physical, emotional, cognitive and social functioning in late life in a nationally representative sample. Respondents were recruited from the population registers of 11 municipalities from three regions in The Netherlands, and were interviewed in their homes by trained persons. In the current study the baseline measurement consisted of data collected in 1995–1996 from participants aged 65–89 years (*N* = 1506) with the aim of ensuring that the proportion of data present at baseline was over 90% on both depression and cognitive measures. Four follow-up measurements were included in this study: 1998–1999 (wave 2), 2001–2002 (wave 3), 2005–2006 (wave 4), and 2008–2009 (wave 5). Fig. 1 presents the number of respondents included in each measurement wave, the attrition rates and the reasons for dropout.

### Instruments

*Depressive symptoms* were assessed using the Centre for Epidemiologic Studies Depression Scale (CES-D; Radloff, 1977). Participants were asked to report how often they experienced certain symptoms of depression in the past week, and their responses were rated using a four-point Likert scale, where 0 = ‘rarely or never’; 1 = ‘some of the time’; 2 = ‘occasionally’; 3 = ‘mostly or always’. CES-D has good psychiatric properties in older adults (Himmelfarb & Murrell, 1983; Hertzog *et al.* 1990; McCallum *et al.* 1995). A cut-off score of 16 can be used to identify persons with major depression (Beekman *et al.* 1997). CES-D was originally posited as having a four-factor structure: depressed affect, positive affect, somatic symptoms and interpersonal difficulties (Radloff, 1977). This factor structure was confirmed in the LASA sample (Beekman *et al.* 1997; Brailean *et al.* 2016). However, the interpersonal difficulties factor may be poorly measured (i.e. it consists of only two items) and it is not in line with the current diagnosis criteria for depression (for a review see Carleton *et al.* 2013). Therefore, in our analyses we only included the subscale scores for positive affect (items: 4, 8, 12, 16), depressed affect (items: 3, 6, 9, 10, 14, 17, 18), and somatic symptoms (items: 1, 2, 5, 7, 11, 13, 20).

*Episodic memory* was assessed using the 15 Words Test, a Dutch version of the Auditory Verbal Learning Test (Rey, 1964). Fifteen words were verbally presented to participants and repeated over three trials. Participants were required to repeat the words they remembered at the end of each trial. The total score on the three trials was used as a measure of immediate recall. After a distraction period of about 20 min participants were asked to name again the words they remembered. This was used as a measure of delayed recall.

*Information processing speed* was assessed in LASA using an adaptation of the Coding Task (Savage, 1984). Participants were shown two rows, the upper one containing characters and the lower one being empty. Participants were asked to name the character in the bottom row which belonged to the character in the upper row and they were instructed to respond to the letter combinations as quickly and accurately as possible. The correct letter combination was presented at the top of the page. The task consisted of three trials and each trial lasted for 1 min. Scores were calculated based on the number of completed combinations. In our analyses we used the total score for the three trials. Because participants were asked to make a verbal response, it is considered that this adapted version of the task measures cognitive speed rather than motor speed.

*Inductive reasoning* was assessed using the Raven Coloured Progressive Matrices (RCPM; Raven, 1995). Participants were shown a drawing from which a section was missing and they were asked to identify the correct missing section from six alternatives patterns presented at the bottom of the page. Raven consists originally of three subsets: A, Ab and B, but only subsets A and B were used in LASA. Each subset consists of 12 items, and both items and subsets show a progressive increase in difficulty. The number of correct responses to each item was used to calculate the total score. The sum score of A and B subsets ranged from 0 to 24.

*General cognitive performance* was assessed with the Mini Mental State Examination (MMSE; Folstein *et al.* 1975), a widely used instrument in epidemiological studies to screen for cognitive impairment and to assess general cognitive functioning in older adults. The MMSE shows satisfactory reliability and construct validity (Tombaugh & McIntyre, 1992). Scores range from 0 to 30 with higher scores indicating better cognitive performance.

### Statistical analysis

First, univariate latent growth curve models (LGCMs), as a function of time in study, were fitted independently to each outcome measure: depressed affect, positive affect, somatic symptoms, immediate recall, delayed recall, processing speed and inductive reasoning. Univariate LGCMs allow for an examination of (1) the initial level of a target outcome (i.e. intercept); (2) its rate of change (i.e. slope) and the form of this change (i.e. linear or nonlinear latent growth trajectory); (3) the association between the initial level of the outcome and its rate of change (e.g. persons who start off with poorer cognitive performance show steeper cognitive decline over time). Various predictors can also be added to these models to show their associations with the initial levels (i.e. intercept) and rate of change (i.e. slope) in the target outcome. In our models the intercept of each target outcome was centred at baseline and a linear form of the latent growth trajectory was tested. Intercepts and slopes of all target outcomes were adjusted for baseline age (in years), gender and education (in years). Age and education were centred at their mean values in order to help with model estimation and with the interpretation of the estimates.

**Fig. 2.**
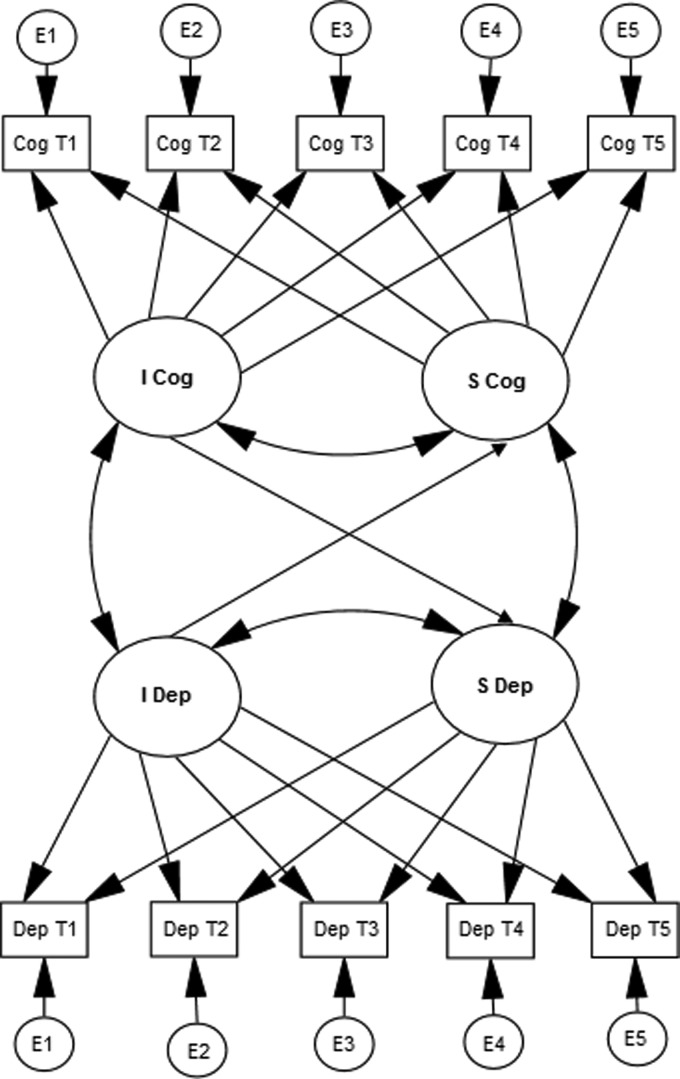
Cross-domain latent growth curve model (LGCM). Cross-domain LGCM illustrating the association between depression and cognitive functioning. Single-headed arrows represent regression effects. Double-headed arrows represent correlations. The intercepts of both depression and cognition are centred at baseline. The slopes of cognition and depression represent changes in these outcomes over five assessment occasions during 13 years of follow-up. The intercepts and slopes of all depression dimensions and cognitive abilities were regressed on relevant covariates. For the sake of clarity, the effect of covariates on the intercept and slope of depression and cognition are not presented in this figure.

After establishing the linearity of the latent growth trajectories and ensuring good model fit in univariate LGCMs, we conducted cross-domain LGCMs to examine the association between each cognitive ability (inductive reasoning, processing speed, immediate recall or delayed recall) and each depression dimension (depressed affect, positive affect and somatic symptoms). On top of the parameters estimated in univariate LGCMs, cross-domain LGCMs estimated associations between: (1) baseline cognitive performance and baseline depression symptoms; (2) baseline cognitive performance and the rate of change in depression symptoms; (3) baseline depression symptoms and the rate of change in cognitive performance; (4) the rate of change in depression symptoms and the rate of change in cognitive performance (Fig. 2). These models were adjusted for baseline age (in years), gender and education (in years).

Sensitivity LGCM analyses were conducted to investigate the association between depression and cognitive functioning in the context of adjustment for potential confounders. Based on previous research (Blazer, 2003; Alexopoulos, 2005; Alzheimer's Disease International, 2014; Baumgart *et al.* 2015; Vassilaki *et al.* 2015), the following confounders were considered as potentially relevant: chronic diseases (i.e. non-specific lung disease, cardiac disease, peripheral arterial disease, diabetes mellitus, cerebrovascular accident or stroke, osteoarthritis or rheumatoid arthritis, cancer, hypertension and a maximum of two other diseases of which symptoms and treatment persisted for at least 3 months), alcohol use (no, middle, and high consumption according to the Netherlands Economic Institute index), exercise (total time spent on physical activities in minutes per day), social network (number of persons with whom the participant is has regular contact), use of antidepressant and anxiolytic medication (user *v*. non-user, based on an inspection of medicine bottles during the medical interview), smoking (current, past, or never smoker). We also repeated the cross-domain LGCM analyses using the total CES-D score instead of domain-specific scores. This additional set of sensitivity analyses included partially adjusted models (i.e. controlling for age, gender and education) and fully adjusted models (i.e. controlling for all the additional confounders mentioned above).

All analyses were conducted in MPlus v. 7.2 (Muthén and Muthén, 1998–2012). Maximum Likelihood Robust (MLR) estimation was used for all models. MLR is robust to non-normality and calculates parameters using both cases with complete data and cases with partially missing data. MLR estimation deals with missing data under the missing at random (MAR) assumption whereby attrition can be related to the observed values of both covariates and outcomes. When the missing data mechanism is MAR, missing data is assumed to be ‘non-informative’ or ‘ignorable’ (Little & Rubin, 1987) and model parameters estimated in the presence of missing data would be similar to the situation in which data had been complete. Model fit was evaluated based on the model χ² with a *p* value >0.05 indicating good model fit (Hu & Bentler, 1999); the comparative fit index (CFI) (Bentler, 1990) and the Tucker–Lewis index (TLI; Tucker & Lewis, 1973) with values >0.90 suggesting acceptable fit, and values >0.95 indicating good fit; the root mean square error of approximation (RMSEA) (Steiger, 1990) with values <0.06 indicating good fit.

## Ethical standards

The authors assert that all procedures contributing to this work comply with the ethical standards of the relevant national and institutional committees on human experimentation and with the Helsinki Declaration of 1975, as revised in 2008.

## Results

Descriptive statistics for our sample are presented in Table 1. Among respondents 51.7% were females, and 13.8% of participants had a MMSE score of ⩽23, indicative of cognitive impairment (Tombaugh & McIntyre, 1992). A CES-D score of ⩾16, indicative of clinically relevant depressive symptoms, was found in 15.8% (*N* = 228) of participants (Berkman *et al.* 1986; Beekman *et al.* 1997). Clinical interviews using the Diagnostic Interview Schedule (Robins *et al.* 1981) were carried out among participants with a CES-D score of ⩾16. Among those interviewed, 37 had a diagnosis of depression in the past 6 months; 21 of them experienced their first episode of depression before age 60 years, and 16 of them experienced their first episode of depression after age 60 years. The age of depression onset was based on participants’ self-reports. The use of antidepressants and anxiolytics was related to cognitive performance on some, but not all cognitive tests (see Supplementary Table S2).

**Table 1.** Descriptive statistics

Continuous measures	*N*	Mean	s.d.	Categorical measures	Category	*N*	%
Age (years)	1506	75.9	6.6	Gender	Male	725	48
Education (years)	1504	8.9	3.3		Female	778	52
Depressed affect	1451	1.8	2.9	Chronic diseases	0	237	16
Positive affect	1408	8.6	3.1		1	454	30
Somatic symptoms	1443	2.9	3.1		2	429	29
Processing speed	1403	68.1	22.0		3	232	15
Inductive reasoning	1425	17.2	4.1		4	112	7
Immediate recall	1457	18.8	6.4		⩾5	38	3
Delayed recall	1457	5.5	3.0	Antidepressants	User	41	3
MMSE	1502	26.5	3.3		Not user	1462	97
Physical activity	1438	40	57	Anxiolytics	User	103	7
Social network size	1447	14.0	8.2		Not user	1400	93
				Smoking	Never	539	36
					Past	680	45
					Current	288	19
				Alcohol	Never	388	26
					Moderate	975	65
					Severe	140	9

MMSE, Mini Mental State Examination.

Physical activity was measured as the total time spent being physically active in minutes per day; social network was measured as the number of persons with whom the participant has regular contact.

Attrition rates and the reason for dropout are presented in Fig. 1. The high attrition rates observed in our study are a typical finding in longitudinal ageing studies. Mortality accounted for over 80% of dropout rates. Other reasons included refusal, ineligibility and lack of contact. Higher dropout rates over the 13 years of follow-up were associated with being older [odds ratio (OR) 1.19, 95% confidence interval (CI) 1.16–1.21], being male (OR 0.56, 95% CI 0.45–0.70), having lower baseline levels of immediate recall (OR 0.88, 95% CI 0.86–0.90), delayed recall (OR 0.78, 95% CI 0.75–0.81), processing speed (OR 0.97, 95% CI 0.97–0.98), and inductive reasoning (OR 0.85, 95% CI 0.83–0.88), as well as having higher baseline levels of depressed affect (OR 1.07, 95% CI 1.03–1.11) and somatic symptoms (OR 1.05, 95% CI 1.01–1.09), and lower levels of positive affect (OR 0.95, 95% CI 0.91–0.98).

### Univariate latent growth models

Table 2 presents results from univariate models adjusted for age, gender and education. Model fit for all univariate models was good and it ranged from CFI = 0.94, TLI = 0.92, RMSEA = 0.07 (90% CI 0.06–0.08) for immediate recall to CFI = 1.00, TLI = 1.00, RMSEA < 0.01 (90% CI 0.00–0.02) for inductive reasoning. The mean of the intercept and slope was statistically significant for all outcome measures, indicating a significant linear change in scores over time. Sample and estimated means for each depression and cognitive outcome measure are presented in Supplementary Table S4. Supplementary Figs S1–S7 present the observed individual scores for each cognitive domain and each depression dimension across time points. Participants showed an increase in depressed affect and somatic symptoms, as well as a drop in positive affect over time. A decline in performance was observed for all cognitive abilities. The intercept and slope of our outcome measures should be interpreted as the initial level and rate of change in the outcome for a person of average age (75.9 years) and average level of education (8.9 years). The variance of the slope for positive affect was not statistically significant, whereas the variance of the intercept and slope for all other outcome measures was statistically significant, indicating that both the initial level and rate of change in depression and cognitive scores varied between individuals. The only significant correlation between intercept and slope was found for processing speed, indicating that participants with higher baseline performance had steeper decline in processing speed over time, which could reflect an effect of regression to the mean. The rate of decline in other cognitive measures was not dependent on the initial level of cognitive performance.

**Table 2.** Estimates for univariate latent growth curve models (LGCMs)

Measure	Mean intercept	Mean slope	Variance intercept	Variance slope	Correlation between intercept and slope	
					*r*	s.e.
Inductive reasoning	17.06	−0.17	7.22	0.02	0.06	0.13
Processing speed	65.60	−1.47	278.4	0.67	−0.22***	0.06
Immediate recall	17.17	−0.42	18.75	0.05	−0.07	0.13
Delayed recall	4.68	−0.18	4.58	0.02	−0.15	0.09
Depressed affect	1.22	0.09	3.96	0.01	−0.08	0.19
Positive affect	9.07	−0.13	4.00	<0.01	−0.01	0.26
Somatic symptoms	2.38	0.15	5.10	0.02	−0.12	0.15

***p < 0.001; the value of the slope reflects the yearly change in the outcome measure; means and variances of all intercepts and slopes are statistically significant, except for the variance of the slope for positive affect; results presented are based on univariate LGCMs for each outcome measure, after adjustment for age, gender and education.

### Cross-domain latent growth models

Table 3 shows results of the cross-domain models. All cross domain models fitted the data well: CFI ⩾ 0.95; TLI ⩾ 0.94; RMSEA ⩽ 0.04 (90% CI 0.04–0.05). Fit indices for each model are presented in Supplementary Table S3. Lower baseline levels of processing speed and immediate recall were significantly associated with higher baseline levels of depressed affect and somatic symptoms, as well as lower levels of positive affect. Higher baseline levels of inductive reasoning were significantly associated with lower depressed affect and lower somatic symptoms. No association was found between baseline levels of delayed recall and baseline levels of depressed affect, positive affect or somatic symptoms. Baseline delayed recall performance predicted a statistically significant steeper increase in depressed affect over time. Initial levels of performance in other cognitive domains were unrelated to rates of change in depressed affect, positive affect or somatic symptoms. Also, initial levels of depressed affect, positive affect or somatic symptoms were unrelated to changes over time in any cognitive domains. In terms of the association between changes in depressive symptoms and changes in cognitive functioning over time, we only found that decline in processing speed performance was related to an increase in somatic symptoms over time.

**Table 3.** Estimates for cross-domain latent growth curve models

	Depression dimension					
	Depressed affect		Positive affect		Somatic symptoms	
Cognitive ability	*β*	s.e.	*β*	s.e.	*β*	s.e.
Processing speed						
I Cog ↔ I Dep	−0.16***	**0.03**	0.13**	0.04	−0.20***	0.04
I Dep → S Cog	0.08	0.08	−0.13	0.08	0.14	0.07
I Cog → S Dep	−0.18	0.10	0.28	0.21	−0.04	0.10
S Cog ↔ S Dep	−0.18	0.12	0.82	0.53	−0.38*	0.16
Inductive reasoning						
I Cog ↔ I Dep	−0.14**	**0.05**	0.07	0.05	−0.18***	**0.05**
I Dep → S Cog	−0.04	0.11	0.08	0.11	0.01	0.11
I Cog → S Dep	−0.12	0.12	0.29	0.26	0.12	0.12
S Cog ↔ S Dep	−0.26	0.17	0.64	0.51	−0.32	0.18
Immediate recall						
I Cog ↔ I Dep	−0.11**	**0.04**	0.10*	**0.05**	−0.12**	**0.04**
I Dep → S Cog	0.16	0.11	−0.06	0.11	0.11	0.10
I Cog → S Dep	−0.18	0.12	0.10	0.21	−0.19	0.12
S Cog ↔ S Dep	−0.32	0.17	0.56	0.43	−0.25	0.17
Delayed recall						
I Cog ↔ I Dep	−0.07	0.04	0.04	0.04	−0.08	0.04
I Dep → S Cog	−0.02	0.10	0.04	0.10	0.01	0.10
I Cog → S Dep	−0.25*	0.11	0.45	0.28	−0.18	0.11
S Cog ↔ S Dep	0.06	0.14	−0.21	0.35	−0.03	0.14

*β*, Standardized estimates; I Cog, intercept of cognitive ability; I Dep, intercept of depression dimension; S Cog, slope of cognitive ability; S Dep, slope of depression dimension; double-headed arrows represent correlations, whereas single-headed arrows represent regression effects; all models are adjusted for age, gender and education.

*p < 0.05, **p < 0.01, ***p < 0.001, statistically significant results are presented in bold.

Sensitivity analyses revealed that adjustment for additional confounders (i.e. number of chronic diseases, physical activity, social network size, alcohol use, use of antidepressant and anxiolytic medication, and smoking) did not change substantive conclusions about the longitudinal associations between depression symptom-dimensions and cognitive abilities (see Supplementary Table S1). The association between processing speed decline and the increase in somatic symptoms remained statistically significant in the fully adjusted models, whereas the effect of baseline delayed recall scores on changes in depressed affect was marginally significant (*p* = 0.05). An additional set of sensitivity analyses using the total CES-D scores revealed that overall depression levels at baseline did not predict the rate of decline in any cognitive ability (see Supplementary Table S5). Higher initial levels of delayed recall predicted an increase in overall depression levels over time, and the effect remained statistically significant in the fully adjusted model. An increase in overall depression levels was associated with decline in immediate recall, processing speed and inductive reasoning in the partially adjusted models, but only the association between processing speed and depression symptoms remained statistically significant in the fully adjusted models.

## Conclusions

Using data from a large nationally representative sample of LASA and a multidimensional approach to late-life depression, this longitudinal study examined associations between depression dimensions and cognitive functioning in late-life. Our findings suggest significant cross-sectional associations between baseline depression dimensions and cognitive performance on processing speed, inductive reasoning and immediate recall tasks. These findings are consistent with evidence that late-life depression co-occurs with impairment in fluid cognitive abilities (e.g. Comijs *et al.* 2001; Butters *et al.* 2004; Sheline *et al.* 2006; Baune *et al.* 2007; Koenig *et al.* 2014). Our longitudinal findings suggest that older adults showed significant decline over time in all cognitive abilities, which is in line with a large body of knowledge suggesting that cognitive decline is part of normal ageing (e.g. Brayne *et al.* 1999; Wilson *et al.* 1999; Park *et al.* 2003). Furthermore, older adults showed an increase in depressed affect and somatic symptoms, as well as a drop in positive affect over time, which is consistent with previous findings suggesting an increase in depressive symptoms in old age (Meeks *et al.* 2011; Sutin *et al.* 2013).

Regarding the effect of baseline cognitive performance on the course of depression symptom-dimensions, our findings indicate that poor delayed recall performance at baseline predicted an increase in depressed affect over time, but it did not predict changes in positive affect or somatic symptoms of depression. Poorer delayed recall performance at baseline predicted also an increase in overall depression symptoms (i.e. total CES-D score), which is consistent with previous reports (Vinkers *et al.* 2004; Jajodia & Borders, 2011). The increase in depressed affect in persons with poor baseline delayed recall may indicate a psychological reaction to perceived memory dysfunction. Older adults may be more likely to notice their memory problems than other cognitive dysfunction (such as slow processing speed). Memory failure may cause difficulties in daily living and related-challenges, which could lead to increasing depressed affect. As our study did not assess subjective memory complaints, the level of insight that participants had about their memory loss remains unknown. However, the effect of initial delayed recall performance on the slope of depressed affect remained statistically significant after adjusting for relevant confounders. An alternative explanation for our finding is that the same neurodegenerative mechanisms may underlie both delayed recall dysfunction and the increase in depression symptoms. According to previous reports, delayed recall measures predict conversion from mild cognitive impairment to Alzheimer's disease better than measures of other cognitive abilities (Gainotti *et al.* 2014), and the risk of conversion is higher among older adults with amnestic cognitive impairment who also present clinical depression, compared to those without depression (Modrego & Ferrandez, 2004). If delayed recall dysfunction and depression symptoms were manifestations of dementia-related neurodegenerative processes, we would have expected delayed recall decline to correlate with an increasing trajectory of depression symptoms. This effect was not found in our study.

Regarding the effect of baseline depression symptom-dimensions on cognitive decline, our findings suggest that baseline levels of depressed affect, positive affect, and somatic symptoms did not predict the rate of cognitive decline. This was also the case for overall depression symptoms (total CES-D score). These findings argue against the hypothesis that depression may be a risk factor for cognitive decline. Our findings conflict with previous reports indicating that higher baseline depression scores predicted accelerated decline in episodic memory (Panza *et al.* 2009; Zahodne *et al.* 2014), processing speed (van den Kommer *et al.* 2013), and general cognitive performance (Gale *et al.* 2012; van den Kommer *et al.* 2013), and are inconsistent with meta-analytic evidence that depressed persons have an increased risk of incident dementia (Ownby *et al.* 2006; Diniz *et al.* 2013; Alzheimer's Disease International, 2014; Cherbuin *et al.* 2015). It is possible that a prospective effect of depression on cognitive decline may have been observed with a shorter follow-up duration than the one used in our study. According to previous reports (Alzheimer's Disease International, 2014; Mirza *et al.* 2014), the prospective effect of depression symptoms on dementia was stronger in studies with shorter follow-up durations, which is consistent with the hypothesis that depression may be a prodromal manifestation of dementia rather than an independent risk factor. Furthermore, it is possible that a clinical diagnosis of depression, rather than subclinical depression symptoms, may flag an increased risk for cognitive decline. In our study only a small percentage of participants had clinically significant depression symptoms (i.e. a CES-D score of ⩾16) or a clinical diagnosis of depression.

Regarding the associations between changes in specific symptom-dimensions of depression and changes in cognitive functioning, our findings suggest that increasing severity of somatic symptoms of depression over 13 years of follow-up showed a specific association with steeper decline in processing speed. This effect remained statistically significant after adjusting for potential confounders. After adjustment for confounders, an increase in overall depression symptoms (i.e. total CES-D score) was only associated with declining processing speed over time. This is inconsistent with previous reports suggesting that changes in processing speed were unrelated to changes in overall depression symptoms in participants aged ⩾55 years (van den Kommer *et al.* 2013). The discrepancy between these findings may be partly explained by the inclusion of older participants in our study (aged ⩾65 years). The concurrent manifestation of somatic symptoms of depression and slow processing speed may be more relevant at advanced ages, but the underlying aetiological mechanisms are yet to be clarified. Previously, Kooistra *et al.* (2015) reported that slower processing speed at baseline was associated with a larger increase in a cluster of somatic and motivational symptoms of depression (compared to mood symptoms) over 7 years of follow-up. The somatic and motivational symptoms of depression assessed by Kooistra *et al.* (2015) were similar to the symptoms assessed in our study with the somatic subscale of CES-D (i.e. appetite disturbance, energy loss, sleep disturbance, concentration problems, psychomotor retardation). Taken together, these findings suggest that somatic and motivational symptoms of depression and processing speed impairment may be clinical manifestation of the same neurodegenerative processes, such as white matter lesions or vascular disease (Alexopoulos *et al.* 1997, 2002; Naarding *et al.* 2005). The specific association between changes in somatic symptoms and changes in processing speed in our study may also reflect the fact that the somatic subscale of CES-D and the processing speed task used in our study measure similar constructs. For instance, some of the items that are part of the somatic subscale of CES-D assess cognitive complaints (i.e. concentration difficulties, feeling that everything is an effort, not being able to get going).

Our findings suggest that an accelerated rate of cognitive decline may be associated with an increase in depression symptoms over time rather than with higher initial depression scores. In particular, the presence of somatic symptoms of depression may indicate a chronic course of cognitive decline which is primarily reflected in slow processing speed. These findings are consistent with evidence that the risk of dementia is particularly high in older adults who experience a chronic course of depression (i.e. an increasing trajectory of depression), but not in those who experience transient depression (i.e. high scores at a particular time point, followed by remission) (Kaup *et al.* 2016; Mirza *et al.* 2016).

Strengths of this study include the large sample size, the long follow-up period, the dimensional approach to late-life depression, and the assessment of several cognitive abilities which are commonly impaired in late-life depression. A first notable limitation of our study is the high attrition rate over the course of the follow-up, largely due to mortality. Participants with more severe depression symptoms and poorer cognitive functioning at baseline were more likely to drop out from the study over the course of the follow-up. The selective loss of more cognitively impaired and more severely depressed individuals over the course of the follow-up may have resulted in an underestimation of the effects found in this study. We dealt with missing data using the maximum likelihood estimation under the missing at random assumption. However, this assumption cannot be verified, and there remains a possibility that findings may differ if data were missing not at random. Second, our findings are only relevant to community dwelling older adults and cannot be generalized to clinical populations. Third, it is possible that our findings may relate to the specific tasks used rather than the cognitive domains being studied. However, we cannot examine this given that only one test was available for each cognitive measure in LASA. Fourth, our models are not adjusted for multiple testing. Although Type 1 error (i.e. the probability of detecting an effect that is not present) could have been reduced by adjusting for multiple testing, this would have been at the expense of the Type 2 error (i.e. failing to detect an effect that is present), and would have reduced the power to detect potentially important effects (Gelman *et al.* 2012). Due to the large number of tests conducted and the correlations among model parameters, traditional methods of correcting for Type 1 error may be overly conservative in the context of our LGCM analyses. Of note, the simultaneous estimation of the effect of baseline depression on cognitive decline and the effect of baseline cognitive performance on the course of depressive symptoms allows to determine relative predictive associations, without implying a true causal effect and without clarifying the aetiological factors that may underlie the observed associations.

In conclusion, our findings do not support the hypothesis that specific depression symptom clusters may predict an increased rate of cognitive decline. However, our findings support a prospective effect of memory function on the course of depressed affect, which may indicate a psychological reaction to poor memory function. This implies that maintaining good cognitive functioning by engaging in memory enhancing activities could help older adults cope with ageing related challenges and protect them against depression. Furthermore, our findings support a synchronous longitudinal association between the course of processing speed performance and the course of somatic symptoms of depression. More research is needed to understand whether somatic symptoms of depression are an early sign of cognitive impairment or a prodromal syndrome of dementia, and whether the early diagnosis and treatment of depression among older adults presenting somatic complaints may improve cognitive outcomes. Future studies could also examine whether older adults with specific trajectory classes (e.g. chronic, remitting, relapsing depression) of depression symptom-dimensions (e.g. depressed affect, positive affect, somatic symptoms) present an accelerated rate of cognitive decline and an increased risk of dementia. A better understanding of the nature, direction and timing of the association between depression symptom dimensions and cognitive functioning in late-life, and of the underlying aetiological mechanisms, could help develop targeted interventions aimed at improving cognitive outcomes among older adults with specific depression symptom profiles.

## Supplementary material

For supplementary material accompanying this paper visit http://dx.doi.org/10.1017/S003329171600297X. click here to view supplementary material
