# Supplementary material for: Longitudinal associations between late-life depression dimensions and cognitive functioning: a cross-domain latent growth curve analysis
Source: Psychol Med. 2016 Nov 11;47(4):690–702. doi: 10.1017/S003329171600297X (PMC5426346; doi:10.1017/S003329171600297X)
Supplement: Supplementary file 1 [file S003329171600297Xsup001.docx]

## **SUPPLEMENTARY MATERIAL**

## LGCM model - syntax example

Data:

File is C:/Users/Anamaria/Desktop/LGCM paper/LGGM model 1.dat;

Variable:

Names are respnr sex aedu cage Ccode Dcode Ecode Fcode Gcode

CSom DSom ESom FSom GSom;

Missing are . ;

Usevariables are Ccode Dcode Ecode Fcode Gcode

CSom DSom ESom FSom GSom sex age edu;

Define:

age = cage - 75.88;

edu = aedu - 8.87;

ANALYSIS:

ESTIMATOR IS MLR;

COVERAGE = 0.10;

Model:

icode scode | Ccode@0 Dcode@3 Ecode@6 Fcode@10 Gcode@13;

iSom sSom | CSom@0 DSom@3 ESom@6 FSom@10 GSom@13;

sSom on icode;

scode on iSom;

iSom with icode;

sSom with scode;

iSom with sSom;

icode with scode;

iSom sSom on sex age edu;

icode scode on sex age edu;

OUTPUT: SAMPSTAT RESIDUAL STANDARDIZED Modindices(All 10) CINTERVAL PATTERNS TECH3 TECH4 TECH1 ;

**Supplementary Table S1.** Estimates for Cross-Domain Latent Growth Curve Models adjusted for confounders

| **Cognitive ability** |  | **Depression dimension** | | | | | |
| --- | --- | --- | --- | --- | --- | --- | --- |
|  |  | **Depressed affect** | | **Positive affect** | | **Somatic symptoms** | |
| **Processing speed** |  | β | S.E. | β | S.E. | β | S.E. |
| I Cog I Dep |  | **-0.11**** | 0.03 | 0.06 | 0.04 | **-0.16***** | 0.04 |
| I Dep S Cog |  | 0.07 | 0.08 | -0.14 | 0.08 | 0.14 | 0.07 |
| I Cog S Dep |  | -0.18 | 0.10 | 0.31 | 0.23 | -0.04 | 0.10 |
| S Cog S Dep |  | -0.17 | 0.13 | 0.97 | 0.76 | **-0.41*** | 0.17 |
|  |  |  |  |  |  |  |  |
| **Inductive reasoning** |  |  |  |  |  |  |  |
| I Cog I Dep |  | **-0.11*** | 0.05 | 0.07 | 0.05 | **-0.16**** | 0.05 |
| I Dep S Cog |  | -0.04 | 0.12 | 0.08 | 0.11 | 0.04 | 0.12 |
| I Cog S Dep |  | -0.14 | 0.12 | 0.29 | 0.26 | 0.10 | 0.13 |
| S Cog S Dep |  | -0.17 | 0.17 | 0.64 | 0.51 | -0.27 | 0.19 |
|  |  |  |  |  |  |  |  |
| **Immediate recall** |  |  |  |  |  |  |  |
| I Cog I Dep |  | -0.07 | 0.04 | 0.04 | 0.05 | -0.08 | 0.04 |
| I Dep S Cog |  | 0.16 | 0.11 | -0.01 | 0.12 | 0.10 | 0.11 |
| I Cog S Dep |  | -0.16 | 0.12 | 0.02 | 0.24 | -0.21 | 0.13 |
| S Cog S Dep |  | -0.30 | 0.17 | 0.83 | 1.06 | -0.22 | 0.18 |
|  |  |  |  |  |  |  |  |
| **Delayed recall** |  |  |  |  |  |  |  |
| I Cog I Dep |  | -0.03 | 0.04 | -0.01 | 0.05 | -0.05 | 0.04 |
| I Dep S Cog |  | -0.01 | 0.11 | 0.05 | 0.11 | 0.03 | 0.11 |
| I Cog S Dep |  | -0.21 | 0.11 | 0.42 | 0.32 | -0.17 | 0.12 |
| S Cog S Dep |  | 0.02 | 0.15 | -0.25 | 0.69 | -0.05 | 0.15 |

*Note:* * p < 0.05; ** p < 0.01; ***p < 0.001; β = standardized estimates; Double headed arrows represent correlations, whereas single headed arrows represent regression effects; All models are adjusted for the number of chronic diseases, physical activity, social network size, use of antidepressant and anxiolytic medication, alcohol use, and smoking; I Cog = intercept of cognitive ability; I Dep = intercept of depression dimension; S Cog = slope of cognitive ability; S Dep = slope of depression dimension.

**Supplementary Table S2.** Differences in baseline cognitive performance according to medication status

|  | **Use of antidepressants** | | |  | **Use of anxiolytics** | | | |
| --- | --- | --- | --- | --- | --- | --- | --- | --- |
|  | Yes  (N=41) | No  (N=1462) |  |  | Yes  (N=103) | No  (N=1400) |  | |
|  | **Means** | | **B** |  | **Means** | | | **B** |
| Immediate recall | 16.5 | 18.8 | -2. 25* |  | 17.2 | 18.8 | -1.61* | |
| Delayed recall | 4.5 | 5.5 | -1.03* |  | 4.7 | 5.5 | -0.81* | |
| Processing speed | 63.3 | 68.2 | -4.93 |  | 62.0 | 68.5 | -6.51** | |
| Inductive reasoning | 16.6 | 17.2 | -0.59 |  | 15.7 | 17.3 | -1.54*** | |
| MMSE | 25.9 | 26.5 | -0.61 |  | 25.9 | 26.5 | -0.61 | |

*Note:* * p < 0.05; ** p < 0.01; ***p < 0.001

**Supplementary Table S3.** Model fit for unadjusted and partially adjusted models

|  | **Depressed affect** | | | | **Positive affect** | | | | **Somatic symptoms** | | | |
| --- | --- | --- | --- | --- | --- | --- | --- | --- | --- | --- | --- | --- |
|  | chi^2^(df) | CFI | TLI | RMSEA  (90 % CI) | chi^2^(df) | CFI | TLI | RMSEA  (90 % CI) | chi^2^(df) | CFI | TLI | RMSEA  (90 % CI) |
| **Processing speed** |  |  |  |  |  |  |  |  |  |  |  |  |
| Unadjusted | 81 (41) | 0.99 | 0.99 | 0.03  (0.02–0.03) | 97 (41) | 0.99 | 0.98 | 0.03  (0.02–0.04) | 106 (41) | 0.98 | 0.98 | 0.03  (0.02–0.04) |
| Partially adjusted | 112 (59) | 0.99 | 0.99 | 0.02  (0.01–0.03) | 121 (59) | 0.99 | 0.98 | 0.03  (0.02–0.03) | 131 (59) | 0.99 | 0.98 | 0.03  (0.02–0.03) |
| **Inductive reasoning** |  |  |  |  |  |  |  |  |  |  |  |  |
| Unadjusted | 42 (41) | 1.00 | 1.00 | 0.01  (0–0.02) | 54 (41) | 0.99 | 0.9 | 0.02  (0–0.03) | 67 (41) | 0.99 | 0.99 | 0.02  (0.01–0.03) |
| Partially adjusted | 56 (59) | 1.00 | 1.00 | <0.01  (0–0.01) | 61 (59) | 1.00 | 1.00 | 0.01  (0–0.02) | 74 (59) | 0.99 | 0.99 | 0.01  (0–0.02) |
| **Immediate recall** |  |  |  |  |  |  |  |  |  |  |  |  |
| Unadjusted | 149 (41) | 0.95 | 0.95 | 0.04  (0.04–0.05) | 194 (41) | 0.94 | 0.93 | 0.05  (0.04–0.06) | 192 (41) | 0.94 | 0.94 | 0.05  (0.04–0.06) |
| Partially adjusted | 175 (59) | 0.96 | 0.95 | 0.04  (0.03–0.04) | 207 (59) | 0.95 | 0.94 | 0.04  (0.03–0.05) | 211 (59) | 0.96 | 0.94 | 0.04  (0.04–0.05) |
| **Delayed recall** |  |  |  |  |  |  |  |  |  |  |  |  |
| Unadjusted | 167 (41) | 0.95 | 0.94 | 0.05  (0.04–0.05) | 194 (41) | 0.94 | 0.93 | 0.05  (0.04–0.06) | 191 (41) | 0.95 | 0.94 | 0.05  (0.04–0.06) |
| Partially adjusted | 191 (59) | 0.96 | 0.95 | 0.04  (0.03–0.05) | 203 (59) | 0.96 | 0.94 | 0.04  (0.03–0.05) | 205 (59) | 0.96 | 0.95 | 0.04  (0.03–0.05) |

*Note:* Unadjusted models do not include any covariates; partially adjusted models include the effects of age, gender and education on cognitive abilities and depression dimensions; this table does not include model fit information for the fully adjusted models (i.e., controlling for age, gender, education, number of chronic diseases, physical activity, social network size, use of antidepressant and anxiolytic medication, alcohol use, and smoking). However, all fully adjusted models fitted the data well and the fit values were similar to the ones in the partially adjusted models.

**Supplementary Table S4.** Sample and estimated means for each outcome measure

|  | **Time** | | | | |
| --- | --- | --- | --- | --- | --- |
|  | **1** | **2** | **3** | **4** | **5** |
| **Sample means** |  |  |  |  |  |
| Depressed affect | 1.8 | 2.0 | 2.4 | 2.5 | 2.5 |
| Positive affect | 8.6 | 8.2 | 7.6 | 7.4 | 7.8 |
| Somatic symptoms | 2.9 | 3.5 | 3.8 | 4.1 | 4.3 |
| Immediate recall | 18.7 | 17.0 | 17.1 | 14.2 | 12.4 |
| Delayed recall | 5.5 | 4.7 | 4.8 | 3.5 | 2.6 |
| Inductive reasoning | 17.2 | 16.5 | 16.2 | 15.5 | 15.0 |
| Processing speed | 67.9 | 64.8 | 60.8 | 54.8 | 47.8 |
| **Model estimated means** |  |  |  |  |  |
| Depressed affect | 1.9 | 2.0 | 2.2 | 2.5 | 2.7 |
| Positive affect | 8.5 | 8.2 | 7.9 | 7.5 | 7.2 |
| Somatic symptoms | 3.0 | 3.3 | 3.7 | 4.2 | 4.5 |
| Immediate recall | 18.8 | 17.4 | 16.1 | 14.3 | 13.0 |
| Delayed recall | 5.5 | 4.9 | 4.3 | 3.5 | 2.8 |
| Inductive reasoning | 17.2 | 16.7 | 16.2 | 15.5 | 15.0 |
| Processing speed | 68.4 | 64.4 | 60.3 | 54.9 | 50.8 |

*Note:* Presented means are based on models adjusted for age, gender

and education

**Supplementary Table S5.** LGCM estimates of the associations between overall depression scores and cognitive abilities

|  | **Overall depression scores** | | | | |
| --- | --- | --- | --- | --- | --- |
| **Cognitive ability** | Partially adjusted  models | |  | Fully adjusted  models | |
|  | β | S.E. |  | β | S.E. |
| **Processing speed** |  |  |  |  |  |
| I Cog I Dep | -0.19*** | 0.03 |  | -0.13*** | 0.03 |
| I Dep S Cog | 0.13 | 0.08 |  | 0.13 | 0.08 |
| I Cog S Dep | -0.15 | 0.10 |  | -0.14 | 0.10 |
| S Cog S Dep | -0.40** | 0.14 |  | -0.43** | 0.14 |
|  |  |  |  |  |  |
| **Inductive reasoning** |  |  |  |  |  |
| I Cog I Dep | -0.15*** | 0.04 |  | -0.12** | 0.05 |
| I Dep S Cog | -0.05 | 0.11 |  | -0.04 | 0.12 |
| I Cog S Dep | -0.09 | 0.12 |  | -0.09 | 0.12 |
| S Cog S Dep | -0.33* | 0.16 |  | -0.28 | 0.16 |
|  |  |  |  |  |  |
| **Immediate recall** |  |  |  |  |  |
| I Cog I Dep | -0.12** | 0.04 |  | -0.07 | 0.04 |
| I Dep S Cog | 0.11 | 0.10 |  | 0.08 | 0.11 |
| I Cog S Dep | -0.19 | 0.12 |  | -0.17 | 0.12 |
| S Cog S Dep | -0.32* | 0.16 |  | -0.31 | 0.16 |
|  |  |  |  |  |  |
| **Delayed recall** |  |  |  |  |  |
| I Cog I Dep | -0.07 | 0.04 |  | -0.02 | 0.04 |
| I Dep S Cog | -0.02 | 0.10 |  | -0.01 | 0.10 |
| I Cog S Dep | -0.28* | 0.11 |  | -0.24* | 0.11 |
| S Cog S Dep | 0.05 | 0.14 |  | 0.01 | 0.14 |

*Note:* *:* * p < 0.05; ** p < 0.01; ***p < 0.001; β = standardized estimates; statistically significant results are presented in bold; double headed arrows represent correlations, whereas single headed arrows represent regression effects; I Cog = intercept of cognitive ability; I Dep = intercept of depression symptoms; S Cog = slope of cognitive ability; S Dep = slope of depression symptoms. Partially adjusted models control only for age, gender and education. Fully adjusted models additionally control for number of chronic diseases, physical activity, social network size, use of antidepressant and anxiolytic medication, alcohol use, and smoking;

**Supplementary Figure S1.** Individual trajectories of processing speed


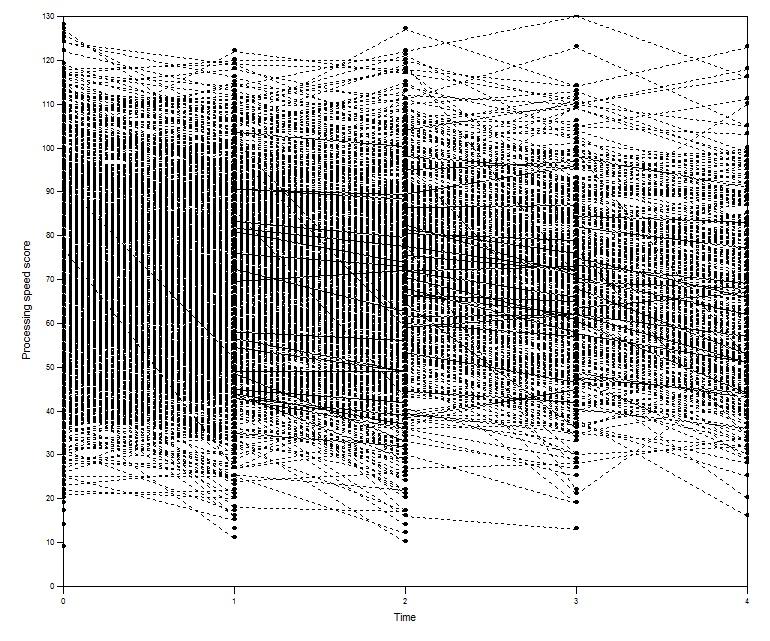


**Supplementary Figure S2.** Individual trajectories of inductive reasoning
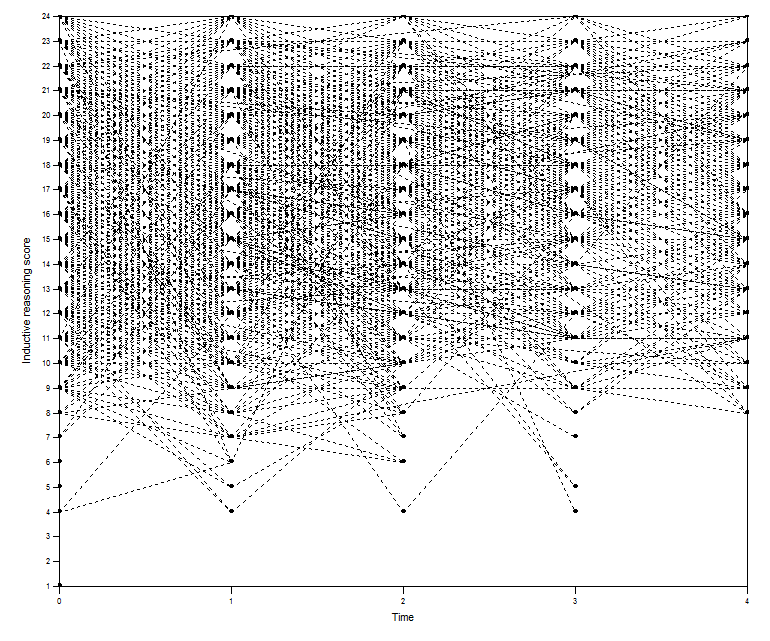


**Supplementary Figure S3.** Individual trajectories of immediate recall


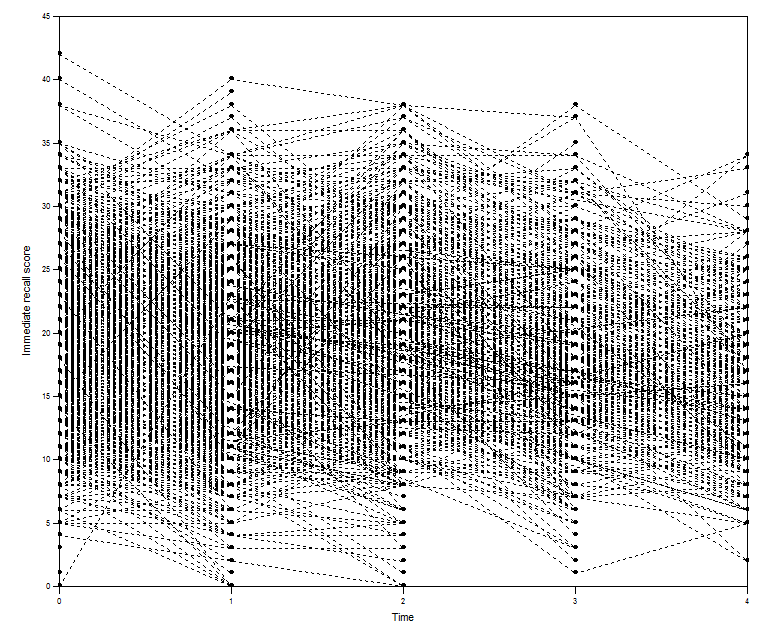


**Supplementary Figure S4.** Individual trajectories of delayed recall
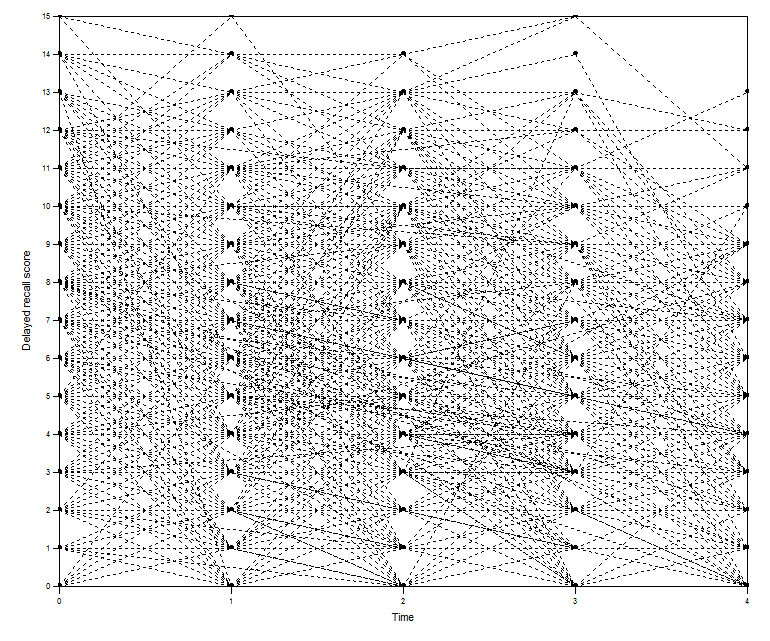


**Supplementary Figure S5.** Individual trajectories of depressed affect


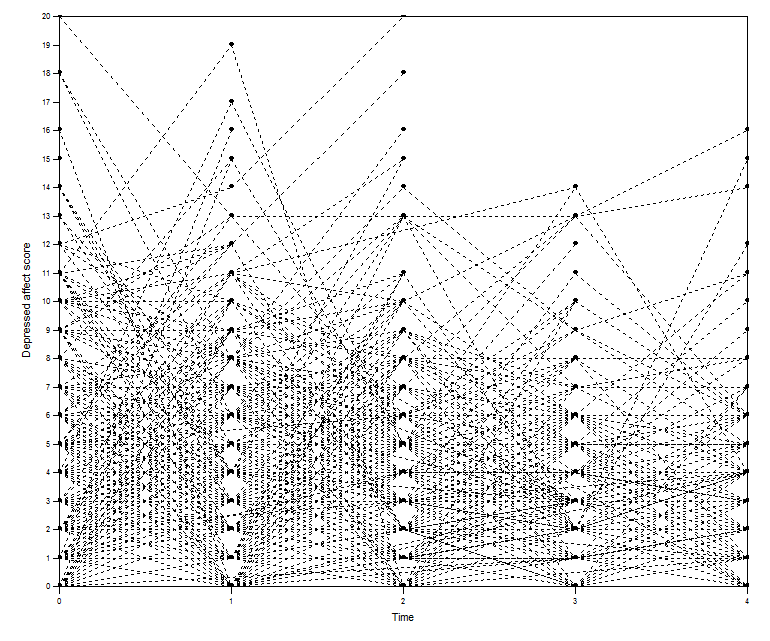


**Supplementary Figure S6.** Individual trajectories of positive affect


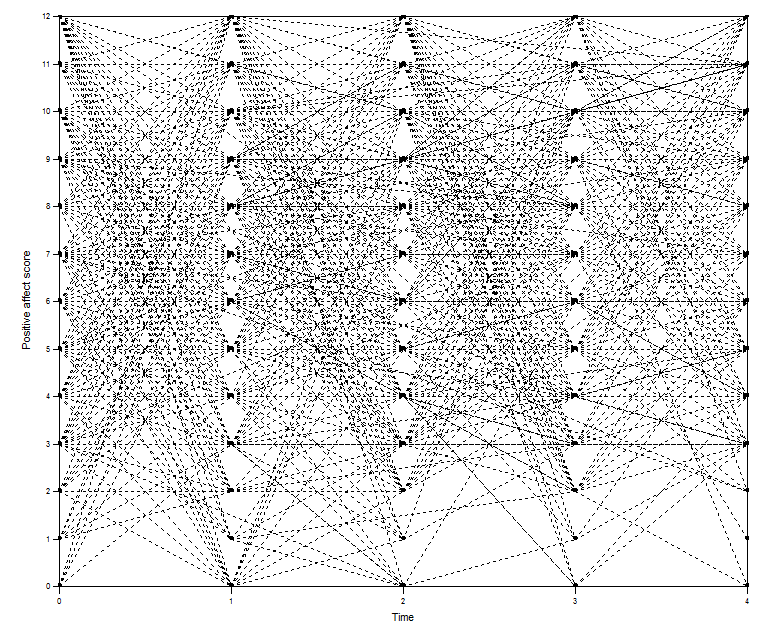


**Supplementary Figure S7.** Individual trajectories of somatic symptoms

**
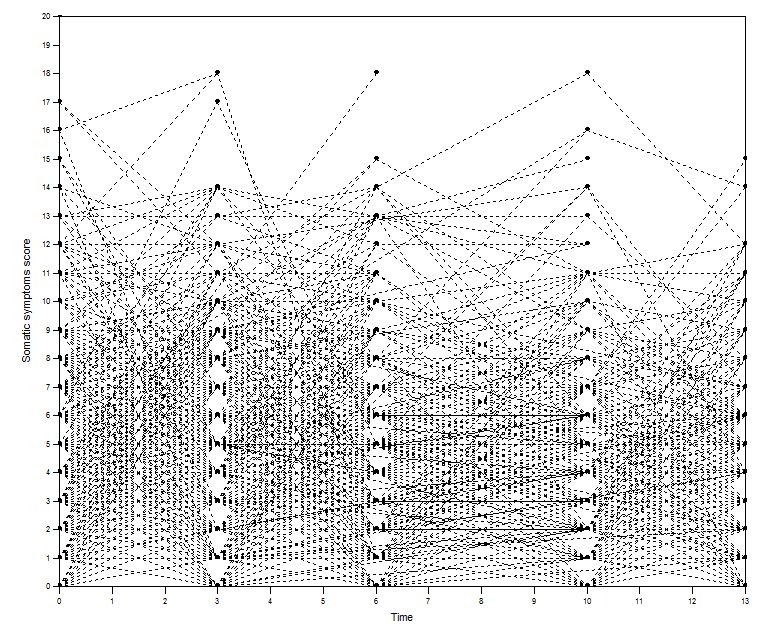
**
